# Prevalence of *Toxocara* Spp. eggs in Public Parks in Tehran City, Iran

**Published:** 2012

**Authors:** H Khazan, M Khazaei, SJ Seyyed Tabaee, A Mehrabi

**Affiliations:** 1 Department of Medical Parasitology and Mycology, Shahid Beheshti University of Medical sciences, Tehran, Iran; 2 Department of Hygiene, School of Public Health, Shahid Beheshti University of Medical Sciences, Tehran, Iran

**Keywords:** *Toxocara*, Iran, Public parks, Soil contamination

## Abstract

**Background:**

The objective of the present research was to determine the frequency of *Toxocara* spp. eggs in soil samples of public parks, in the city of Tehran, Iran.

**Methods:**

A total of 600 soil samples were taken from 120 parks between Aprils to November, 2008. Soil samples were collected from 5 distinct sites in the parks. The samples were washed with saline solution and the collected sediment from each park were equally divided and examined by floatation and Petri dish methods for *Toxocara* eggs.

**Results:**

Ten percent were contaminated with *Toxocara* spp. eggs. The number of observed *Toxocara* eggs in each microscopic field was varied from 1-3. No significant differences were observed between floatation and Petri dish methods.

**Conclusion:**

Our public parks showed a high risk of toxocariasis and the need for preventive studies.

## Introduction

*Toxocara canis* and *T. cati* are common intestinal parasites of dogs and cats. The soil contamination with eggs of these parasites is an important etiological factor in *Toxocara* infection of people.

Human beings become infected by ingesting infective eggs (1–2). Human infection with toxocariasis is mostly asymptomatic in the most individuals. However, the immune system unable to control larvae migration into liver, in these cases, otherwise involvement of central nervous system and/or eye can be occurring. Among children, the age groups most affected by severe clinical symptoms of larva migrant's syndrome are toddlers 1-3 years (3). The prevalence of *Toxocara* eggs infected soil is reported from 0.8% in Costa Rica to 97.5% in Greece (6, 7). There are few studies in Iran on the prevalence of *Toxocara* eggs in public parks. The purpose of this study was to point out the prevalence of contamination public parks with *Toxocara* spp. eggs in Tehran.

## Materials and Methods

From April to November 2008, 120 parks were selected from 19 different zones of Tehran and soil samples were taken. Five soil samples, each 100g were collected (from north, south, west, east and central of each park). After pooling the samples of each park, a 500g sample was washed with saline solution into buckets through a set of 2 sieves having pore widths of 250µm and 150µm.

The water collected in the bucket was left to sediment for 1-2 hours. The sediment from each park were equally divided and examined by floatation method with saturated salt solution (8) and Petri dish plate for *Toxocara* eggs. The sediment in petri dishes were diluted in saline and examined under stereomicroscope for the presence of *Toxocara* eggs.

## Results

We studied 120 parks in Tehran for *Toxocara* eggs contamination. Results are seen in Table 1.

**Table 1:** The contamination of soil parks in Tehran city by *Toxocara* eggs in 2008. Numbers of eggs in soil sample were varied from 1-3 eggs (Continued next column)

Zone	Number of studied park	Number of infected park
1	11	7
2	11	3
3	4	1
4	16	1
5	8	1
6	3	0
7	3	0
8	6	1
9	8	2
10	5	0
12	2	1
13	3	0
14	11	3
15	9	1
16	2	0
17	7	0
18	6	0
19	3	1
20	2	0
Total	120	22

**Fig. 1:**
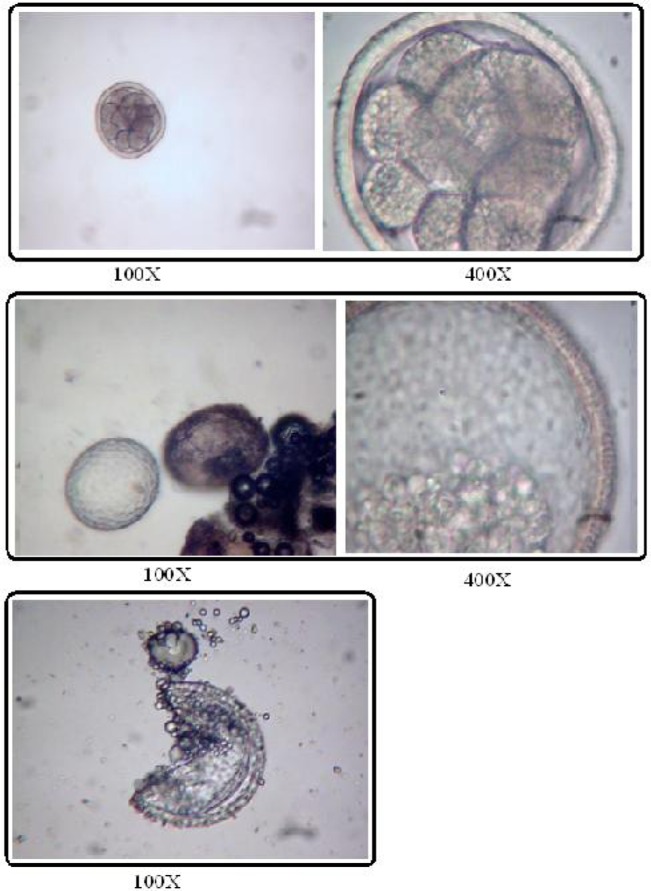
Toxocara canis ova detected in floatation and Petri Dish methods (Source: Authors)

**Fig. 2:**
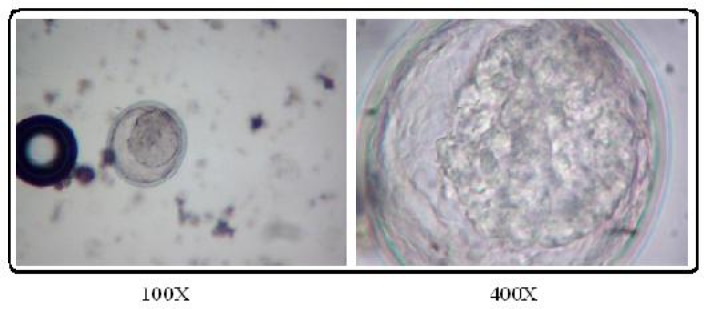
*Toxascaris* ova detected in Petri Dish method (Source: Authors)

The prevalence of contamination was 18.3% and 10% for ascarid eggs and *Toxocara* eggs respectively. *Toxocara* spp. eggs (Figs. 1 and 2) were prevalent parasite (51.92%), followed by *Toxascaris leonina* (40.39%) (Table 2).

**Table 2:** Kinds of parasite detected in the soil parks in Tehran

Kind of parasite	No. of parasite in parks	Infection (%)
*Toxocara* eggs	27	51.92
*Toxascaris* eggs	21	40.39
Ancylostomatidae eggs	2	3.85
*Isospora* oocyst	1	1.92
*Eimeria* oocyst	1	1.92
Total	52	100

As shown in Table 3, no significant differences were found between methods of salt saturated solution and Petri dish for eggs detection.

**Table 3:** Detection of ascarid eggs in soil parks of Tehran by floatation & Petri dish methods in 2008

Floatation			
Petri dish	+	-	Total
+	2	3	5
-	17	98	115
Total	19	101	120

## Discussion

*Toxocara* spp. is the most common nematodes in dogs and cats. Human *Toxocariasis* develops by ingesting of embryonated eggs in contaminated soil. All individuals are susceptible to contamination; however, children play in the parks more than the adults.

We found eggs of *Toxocara* spp. from 12(10%) out of the 120 public parks researched by the laboratorial techniques.

The contamination found in Tehran is lower than from many cities in world as: Thessalonki/Greece (97.5%), Franfurt/Germany (87.1%), Tokushma/Japan (63.3%), Khorramabad/Iran (63.3%), Sao Paulo /Brazil(60%), Petaling jaya/Malaysia (54.5%), Havana/Cuba(42.2%), Ankra/Turkey(30.6%), Konya/Turkey(25%), Kansas/USA(20.6%) and Aydin/Turkey(18.9%) (7, 9–18).

The contamination in our study was higher than the contamination found in Buenos Aires/Argentina (7.2%), London/UK (6.3%), Shiraz /Iran (6.3%), Dublin/Ireland (5.6%), Urmia/Iran (3.9%), Resistencia /Argentina (1.3%) and Muracia/Spain (1.2%) (19–25).

All these results are different because many factors can be effective on this topic, from socio cultural to geographical parameters and examination methods. Therefore, we can not exactly compare all such studies. Toxocariasis infection in dogs and cats in Iran were reported from 10-51.6% (4–5) and 13-52.7%, respectively (26, 27).

In four serodiagnosis studies of toxocariasis in Iranian children have been shown as, 10 cases in Iran, 25.6% from Shiraz, south of Iran, 5.3% in west of Iran and 2.7% from northwest of Iran, respectively (5, 28–30).

In spite of the light contamination rate and low number of eggs found in this study it should be kept in mind that children always take a risk of visceral larva migrants while playing in contaminated playgrounds. For this reason, preventive measures should be implemented. These could include health education of the public health, good personal hygiene practice, control of stray dogs and cats.
